# Natural Selection on Female Life-History Traits in Relation to Socio-Economic Class in Pre-Industrial Human Populations

**DOI:** 10.1371/journal.pone.0000606

**Published:** 2007-07-11

**Authors:** Jenni E. Pettay, Samuli Helle, Jukka Jokela, Virpi Lummaa

**Affiliations:** 1 Section of Ecology, Department of Biology, University of Turku, Turku, Finland; 2 Eawag, Swiss Federal Institute of Aquatic Science and Technology, Dübendorf, Switzerland; 3 Institute of Integrative Biology (IBZ), Swiss Federal Institute of Technology (ETH Zürich), Zürich, Switzerland; 4 Department of Animal and Plant Sciences, University of Sheffield, Sheffield, United Kingdom; University of Cambridge, United Kingdom

## Abstract

Life-history theory predicts that resource scarcity constrains individual optimal reproductive strategies and shapes the evolution of life-history traits. In species where the inherited structure of social class may lead to consistent resource differences among family lines, between-class variation in resource availability should select for divergence in optimal reproductive strategies. Evaluating this prediction requires information on the phenotypic selection and quantitative genetics of life-history trait variation in relation to individual lifetime access to resources. Here, we show using path analysis how resource availability, measured as the wealth class of the family, affected the opportunity and intensity of phenotypic selection on the key life-history traits of women living in pre-industrial Finland during the 1800s and 1900s. We found the highest opportunity for total selection and the strongest selection on earlier age at first reproduction in women of the poorest wealth class, whereas selection favoured older age at reproductive cessation in mothers of the wealthier classes. We also found clear differences in female life-history traits across wealth classes: the poorest women had the lowest age-specific survival throughout their lives, they started reproduction later, delivered fewer offspring during their lifetime, ceased reproduction younger, had poorer offspring survival to adulthood and, hence, had lower fitness compared to the wealthier women. Our results show that the amount of wealth affected the selection pressure on female life-history in a pre-industrial human population.

## Introduction

Life-history theory predicts that resource availability plays a central role in the optimization of individual life-history strategies [1]. Access to resources is vital for an individual's survival and reproduction, and ultimately for its evolutionary success. Environments with plentiful of resources should therefore be associated with, for example, earlier age at maturity and higher reproductive success and survival. In environments where the energy available is more limited, the expression of optimal combination of traits should be more constrained. Limited availability of resources should also promote trade-offs between fitness-related traits that compete for the same resource pool (e.g. somatic maintenance vs. reproductive investment), and thus the optimal within-individual allocation of resources is likely to change across resource regimes [1]–[3]. These classical predictions of the life-history theory emphasize that if individuals within the population experience variation in resource availability, this may shape the resource allocation strategies and trade-offs observed in the population.

Among-individual variation in resource availability has been suggested to affect trade-offs between human life-history traits, highlighting the importance of resource availability on human life-history evolution. In general, younger age at first and advanced age at last reproduction as well as the larger number of offspring born has been shown to be the most important components determining female fitness in both historical Sami [4]–[5] and contemporary Western populations [6]. In addition, long maternal post-menopausal lifespan was recently shown to increase the long-term fitness (i.e., the number of grandchildren born) of pre-industrial Finnish mothers, since long-lived grandmothers were able to improve the reproductive success of their offspring [7]. However, unequivocal conclusions concerning how natural selection has affected human life-history trait evolution are difficult at present. The difficulty arises partly because an individual's access to resources depends on the complex social hierarchies inherent to human societies that are difficult to document *post-hoc,* and partly because of our limited understanding of how natural selection varies in relation to variation in resource availability. Yet, human life-history evolution is not the only research area where information on the relationship between resource availability and the strength of natural selection and trait evolution is needed. Such information is relevant for any species where populations are socially structured or which show temporal and spatial heterogeneity in resource availability, as these factors can lead to differential selection regimes. Currently, we have no data to evaluate how important or general such phenomena are.

In addition to resource-dependent trade-offs, life-history theory predicts that in iteroparous organisms an increase in extrinsic mortality selects for optimal life history that shifts towards earlier reproduction, and higher reproductive effort [8]. Such a shift to earlier reproduction has been observed, for example, in some intensively harvested fish species [9] and in natural populations of guppies (*Poecilia reticulata*) [10]–[11]. In historical human populations, infectious diseases and malnutrition were the two main causes of mortality [12]–[13]. Consequently, survival chances were coupled with wealth; even in modern developed countries, individuals with low socio-economic status have, on average, lower life-expectancy [14]–[15]. In historical Finland, famines resulting from poor crop yields were especially severe for poor landless people, who were forced to beg for food [16]. While such people were not necessarily dying from hunger, mortality from infectious diseases was likely higher among these people due to poorer housing and hygiene [17]. Walker et al. [18] studied several small-scale human/hunter-gatherer societies to distinguish between the effects of energetic constraints and selection on child and juvenile growth rates, and age at menarche and first reproduction. In societies with larger and taller adults (indicators of good nutrition), child growth rates were faster and age at menarche and first reproduction occurred earlier. However, faster child to juvenile (from 3 to 10 years) growth rates and earlier ages at menarche and age at first reproduction were related to higher juvenile mortality in these populations. In other words, at the population level selection can promote earlier maturation and reproduction, but at the individual level resource availability may constrain such an optimal allocation to be expressed.

Previous studies considering how resource availability affects selection in humans have mainly focused on understanding the demographic transition: the association between reduced family sizes with increasing wealth in industrialized countries [19]. Much less is currently known concerning how resource variation affects the strength and direction of selection on human life history, despite this being one of the basic premises of life-history theory. One example is a study by Lummaa et al. [20]–[21], who demonstrated that in pre-industrial Finland selection favoured heritable dizygotic twinning in populations enjoying a predictable food supply, whereas twinning was selected against in populations suffering from frequent famines. These results suggest that the differing selection pressure on multiple births led to significant differences in twinning rates between populations with differing access to resources [20]. Further evidence that resource availability may affect selection on life-history traits in humans comes from studies on historical Germans and Swedes [22]–[23]. In these populations, a negative relationship (i.e., trade-off) between parity and post-menopausal lifespan existed among poor landless women only, whereas in wealthier farmer and smallholder women, the relationship between parity and post-menopausal lifespan was actually positive.

In summary, earlier studies have documented that resource availability may affect the expression of human life-history traits, and alter the trade-offs between them. Studies have also shown how natural selection may have shaped human life-history traits. Furthermore, recent studies have revealed relatively high heritability of key life-history traits in human populations, including those studied here, suggesting that also rather rapid evolutionary responses may have been possible [6], [24]. In this study, we explicitly address the importance of population subdivision by wealth (an estimate of individual access to resources, divided to poor, middle and rich classes) on the expression of female life-history traits and on the natural selection affecting them. Our main aims are (i) to investigate the age-specific survival probabilities of women in each wealth class, (ii) to analyse whether the opportunity, strength and direction of natural selection on key female life-history traits varies with respect to variation in resource availability, and (iii) to compare the mean values of these female life-history traits with respect to wealth class in pre-industrial women born in five Finnish populations during 1702-1863. We examine the age-specific survival of women according to their wealth class to demonstrate the different mortality regimes between wealth classes. We designed the selection analyses to investigate, for example, the prediction that women in the poorest wealth class facing the highest mortality rates should experience the strongest selection on early reproduction, while stronger selection for delayed reproduction should be seen among women with less limited resource availability and higher survival rates. Because correlations between the life-history traits may exist, it is crucial to consider selection on several life-history traits simultaneously [25]. We thus take an advantage of path analysis to model and estimate the strength of natural selection on complex female life history [26]. Furthermore, the measurement of direction and magnitude of selection is most reliable when data on lifetime fitness are available [25]. We therefore use a detailed data set that includes records of full life histories and lifetime reproductive success of women belonging to different wealth classes. Our study period precedes a period of more liberal economics and improvements in healthcare that reduced the mortality and fertility rates in this population [27]. Such data are well suited for analyses aiming to determine the importance of resource variation for selection on key female life-history traits.

## Results

### Wealth class and lifetime survival

**Figure 1:**
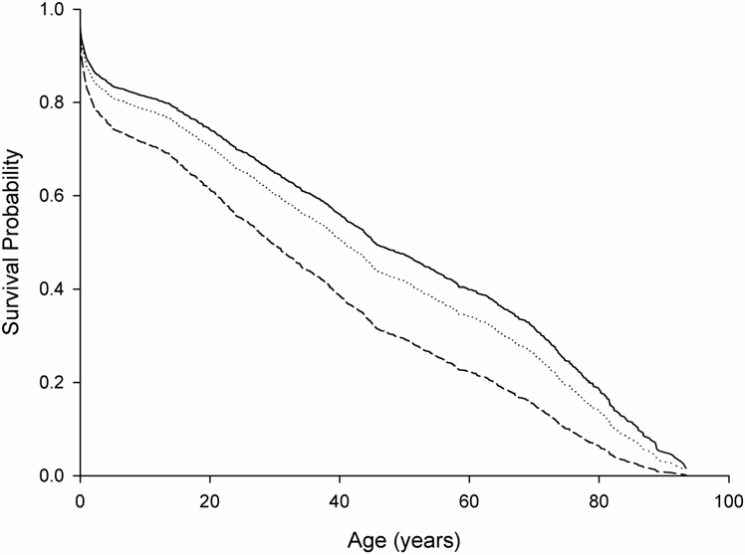
Estimated Kaplan-Meier survival curves for pre-industrial Finnish women belonging to the Rich (solid line), the Middle-class (dotted line), and the Poor (slashed line) parental wealth class, while adjusting for study parish and birth cohort.

Cox survival analysis indicated that mortality rates differed between women belonging to different parental wealth classes (see methods for more details on those definitions, *n* = 2038, *χ^2^_2_* = 25.92, *P*<0.0001, controlling for study parish and birth cohort with a linear model; Fig. 1). Women from the Poor families had lower survival rates compared to the Rich (*n* = 1289, *χ^2^_1_* = 21.53, *P*<0.0001) and the Middle-class (*n* = 850, *χ^2^_1_* = 9.81, *P* = 0.002) families throughout their lifespan, and women from the Rich families had higher survival than women from the Middle-class families (*n = *1937, *χ^2^_1_* = 9.54, *P* = 0.002; Fig. 1). These results emphasize the harsh living conditions experienced by the mothers of the poorest wealth, and demonstrate the different mortality regimes experienced by the mothers in different wealth classes.

### Wealth class and selection on life-history traits

First, we documented the overall constraint on selection for female life-history traits between the wealth classes by estimating the opportunity for selection, which measures variance in fitness [25], [28], for each wealth class. The opportunity for total selection (I_LRS_) differed significantly between the wealth classes (Table 1). The opportunity for total selection was the highest among the mothers of the poorest wealth class and decreased gradually towards the wealthier mothers (Table 1). Estimating the opportunity for selection on two major female fitness components, i.e., on fecundity (I_fec_) and longevity (I_long_), suggested similar trends as above between the wealth classes, but these differences were not statistically significant (Table 1).

**Table 1:** Wealth class-specific estimates of opportunity for selection on total selection. (fitness, *I_LRS_*), fecundity (*I_fec_*), and longevity (*I_long_*) in pre-industrial Finnish women.

	Rich	Middle-class	Poor	χ^2^	P
*I_LRS_*	0.287	0.357	0.660	5.99	0.003
*I_fec_*	0.197	0.266	0.504	2.80	0.06
*I_long_*	0.059	0.066	0.084	2.69	0.3

**Figure 2:**
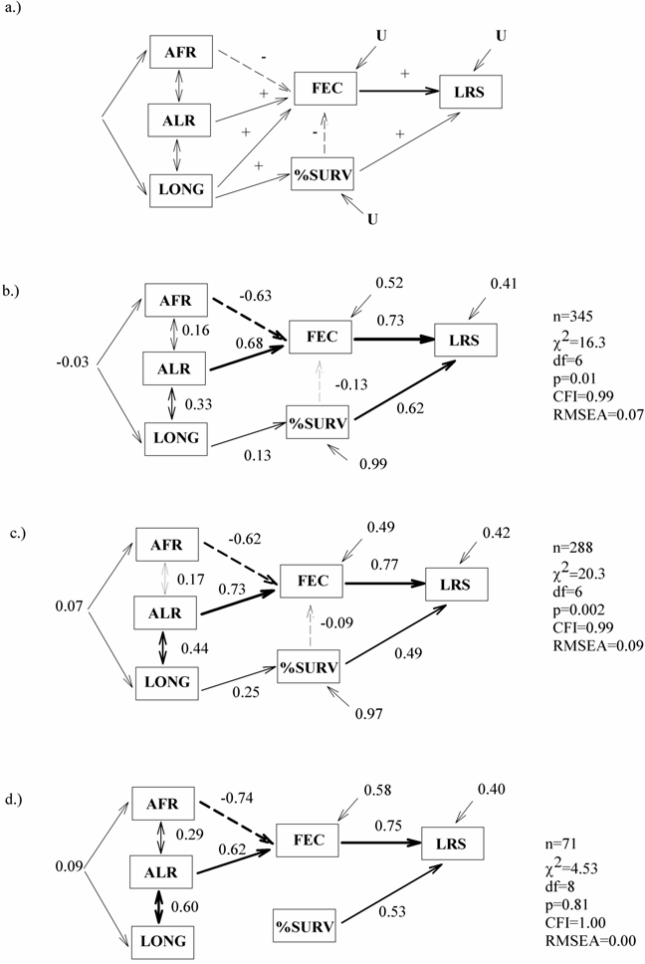
Initial theoretical path model (a) and the final model for the Rich (b), the Middle-class (c), and the Poor (d) wealth class. These models describe linear selection gradients (i.e., standardized partial regression coefficients) of age at first reproduction (AFR), age at last reproduction (ALR), longevity (LONG), fecundity (FEC), and offspring survival (%SURV) on lifetime reproductive success (LRS) of historical Finnish mothers. Single headed arrows represent assumed causal relationships and double-headed arrows non-causal correlation between two variables. Positive selection gradients are given in solid lines and negative selection gradients in dashed line. Thickness of the arrows represents the magnitude of that association. Statistically non-significant selection gradients are omitted from the final path models. U denotes to the error variance in dependent variables not explained by antecedent variables in the model.

Second, we studied the strength and direction of natural selection on female life-history traits using the path analysis. These wealth class-specific path models demonstrated that across the wealth classes fecundity was the most important female life-history trait (Table 3, Fig. 2). The more offspring women gave birth to, the higher was their fitness. Not surprisingly, offspring survival to adulthood was also under strong positive selection in all of the wealth classes. In the Rich and the Middle-class women, later age at last reproduction was more important for fitness than earlier age at first reproduction, whereas in the Poor earlier age at first reproduction was more important (Table 2). Furthermore, selection for longer lifespan was strongest in the Middle-class women and lowest among the Poor women. Correlations between the age at first and last reproduction and longevity were strongest in the Poor women. Contrary to the Rich and Middle-class, longevity was not directly associated with offspring survival in the Poor class (Fig. 2, Table 3). We also found offspring survival to have a negative effect on fecundity in the Rich and the Middle-class, but not in the Poor (Fig. 2). In sum, our results reveal substantial differences in fitness payoffs for several maternal key life-history traits between the wealth classes.

**Table 2:** Estimates of the strength of natural selection (selection differential, and its components, direct and indirect selection) on female life-history traits in the 18th-19th century Finland.

Wealth class	Life-history trait	Direct selection	Indirect selection	Selection differential
**Rich**	Fecundity	0.73		0.73
	% offspring surviving	0.62	−0.09	0.53
	Age at last reproduction	0.50	−0.04	0.45
	Age at first reproduction	−0.46	0.08	−0.38
	Longevity	0.07	0.18	0.25
**Middle-class**	Fecundity	0.77		0.77
	% offspring surviving	0.49	−0.07	0.42
	Age at last reproduction	0.56	−0.05	0.52
	Age at first reproduction	−0.48	0.10	−0.37
	Longevity	0.12	0.21	0.34
**Poor**	Fecundity	0.75		0.75
	% offspring surviving	0.53		0.53
	Age at last reproduction	0.47	−0.16	0.30
	Age at first reproduction	−0.56	0.13	−0.42
	Longevity		0.23	0.23

**Table 3:** Results of GLMMs investigating interactions between wealth class and female life-history traits.

*A. WEALTH CLASSES POOLED*	F_NDF,DDF_	P
**FEC∼AFR+ALR+%SURV**		
AFR×W	4.49_2,669_	0.01
ALR×W	6.27_2,681_	0.002
%SURV×W	4.68_2,680_	0.01
**LRS∼FEC+%SURV**		
FEC×W	2.02_2,688_	0.13
%SURV×W	57.79_2,690_	<.0001
B. MIDDLE-CLASS vs. POOR		
**FEC∼AFR+ALR+%SURV**		
AFR×W	0.15_1.341_	0.7
ALR×W	7.01_1,341_	0.009
%SURV×W	3.30_1,341_	0.009
**LRS∼FEC+%SURV**		
%SURV×W	28.05_1,448_	<.0001
C. RICH vs. POOR		
**FEC∼AFR+ALR+%SURV**		
AFR×W	4.14_1,394_	0.04
ALR×W	12.24_1,396_	0.0005
%SURV×W	6.71_1,397_	0.01
**LRS∼FEC+%SURV**		
%SURV×W	90.52_1,400_	<0.0001
D. RICH vs. MIDDLE-CLASS		
**FEC∼AFR+ALR+%SURV**		
AFR×W	7.93_1,579_	0.005
ALR×W	1.77_1,600_	0.2
%SURV×W	3.17_1,612_	0.08
**LRS∼FEC+%SURV**		
%SURV×W	41.02_1,614_	<.0001

(A) pooled data, and (B-D) pairwise comparisons between the wealth classes. The table shows results for two models in each of the above cases: first, effects of interactions between the wealth class (W) and age at first reproduction (AFR), age at last reproduction (ALR) and longevity (LONG) on fecundity (FEC) and, second, the effects of interactions between wealth class and fecundity and offspring survival (%SURV) on lifetime reproductive success (LRS). The main terms of these variables are omitted from the table for simplicity.

Third, we studied differences in the path coefficients between different wealth classes by analysing interactions between the wealth class and selected path coefficients using general linear mixed models (GLMMs). These analyses showed that age at first and last reproduction and offspring survival to adulthood affected female fecundity differently among the wealth classes, whereas fecundity had similar effects on fitness across the wealth classes (Table 2). Pairwise comparisons between the wealth classes revealed that most of the statistically significant differences in path coefficients were between the Poor and the other two wealth classes (Table 2). Note, however, that these comparisons have only limited value here, because our deduction on the strength of natural selection on female life history is based on selection differentials, not on single path coefficients. It is not possible to test the statistical significance of a selection differential, if it involves intermediate steps to fitness or indirect selection [26].

### Differences in life-history traits between the wealth classes

Variation in maternal life-history traits by wealth class is summarized in Table 4. In the studied populations, marriage rates were high, as only 8% of women surviving to age of 20 failed to marry in their lifetime. Parental wealth class did not affect a female's probability of marriage, or her age at marriage (Table 4). We also analysed a female's age at marriage in relation to the wealth of her husband, and found that rich men married significantly (two years younger than the Middle-class and five years younger than the Poor) younger women (Table 4).

**Table 4:** Least Square means (± SE), sample sizes, and the results of statistical tests for the differences in life-history trait means in relation to wealth class.

Trait	Rich		Middle-class		Poor		F_NDF,DDF_	P
	lsmean±SE	n	lsmean±SE	n	lsmean±SE	n		
Lifetime reproductive success	4.27±0.12	345	3.5±0.15	288	2.01±0.26	71	36.13_2,289_	<.0001
Fecundity	7.38±0.27	345	6.33±0.28	288	4.60±0.38	71	33.71_2,284_	<.0001
Age at marriage*	25.54±0.27	506	25.94±0.36	295	25.65±1.08	29	0.39_2_,_385_	0.7
Age at marriage	23.00±0.53	345	25.15±0.53	288	28.21±0.78	71	34.41_2,250_	<.0001
Age at first reproduction	25.48±0.41	345	27.43±0.42	288	28.87±0.71	71	10.53_2,117_	<.0001
Age at last reproduction	39.46±0.54	345	39.05±0.57	288	36.93±0.77	71	6.34_2,285_	0.002
Lifespan	62.42±1.60	345	60.64±1.67	288	57.60±2.29	71	2.71_2,285_	0.07
Time to birth after marriage	18.20±1.32	321	17.09±1.55	227	14.29±3.59	41	0.57_2,217_	0.6
Offspring survival	0.60±0.02	345	0.58±0.02	288	0.49±0.03	71	4.82_2,289_	0.009
Marriage probability*	0.89±0.01	532	0.88±0.02	328	0.94±0.05	31	1.152	0.6
Number of grandchildren	15.77±0.68	208	11.16±0.90	156	6.08±2.00	24	15.49_2,381_	<.0001

* Analyses where parental wealth class has been a factor instead of a woman's marital wealth class

Rich women started reproducing earliest, had the highest fecundity and reproductive success and, finally, the highest number of grandchildren (Table 4). Pairwise comparisons showed that age at first reproduction was significantly (two and three years, respectively) earlier in the Rich compared to the Middle-class (*t_117_* = −3.36, *P* = 0.003, adjusted by Tukey's test) and to the Poor (*t_117_* = −4.12 *P* = 0.0002), but that age at first reproduction did not differ between the Middle-class and the Poor (*t_117_* = −1.75 *P* = 0.2). Age at last reproduction did not differ significantly between the Rich and the Middle-class mothers (*t_285_* = −0.90, *P* = 0.7), but the Poor had their last child, on average, two years earlier than the Rich (*t_285_* = 3.53, *P* = 0.001) and Middle-class women (*t_285_* = 3.09, *P* = 0.006; Table 4). There was also a gradient for lower offspring survival from the Rich to the Poor (Table 4), with 60 percent of the Rich mothers' children surviving to adulthood while less than half of the Poor mothers' children survived. Mean length in months from marriage to first childbirth did not differ between wealth classes, when those mothers that gave birth before marriage were excluded (*n* = 60, 9%) (Table 4). Mean maternal lifespan did not differ between the wealth classes, but one should note that this analysis only included women who had at least one child and had therefore survived until childbearing age. Finally, the Poor mothers had only half the number of grandchildren than the Rich mothers (Table 4), indicating that the constraints on maximising various life-history components among the Poor women resulted in reduced long-term fitness.

## Discussion

To our knowledge, this is the first study to show that resource availability influenced natural selection on female life-history traits in pre-industrial humans. In agreement with the life-history theory, the opportunity for total selection, the strength of natural selection on life-history traits, and trait means differed between women belonging to different wealth classes in the studied pre-industrial Finnish populations. We found higher opportunity for selection and stronger selection on earlier age at first reproduction among the mothers of the poorest wealth class compared to wealthier mothers. In contrast, selection favoured older age at reproductive cessation in the mothers of higher resource availability compared to poorer mothers. These results are in line with the prediction that selection should favour early reproductive effort in conditions where mortality is high, as women in the poorest wealth class suffered from the highest age-specific mortality. In the Middle-class mothers selection also favoured longer lifespan more than in the other wealth classes. Compared to the poorer mothers, Rich women started their reproduction earliest, had the highest fecundity and reproductive success and, finally, the highest number of grandchildren born.

These results support the previous findings suggesting that parity, together with age at first and last reproduction are among the most important components of fitness in historical women [4]–[5]. Our findings add to these results by showing that the strength of selection on these traits may depend on the resources available to women. For example, in the Poor mothers living in the most resource limited conditions, earlier age at first reproduction was under stronger selection than later age at last reproduction, whereas the opposite was true for wealthier mothers. These results are in accordance with the life-history theory that predicts stronger selection for early reproduction in conditions where mortality rate is high and late reproductive career less likely [28]. Even though the Poor women would have benefited the most from early reproduction, they had the latest mean age at first reproduction. There are at least three possible explanations for this. First, the Poor women may have had a disadvantage at the marriage market [29]. In these data, parental wealth class did not affect the marriage age of women. Instead, richer men seemed to marry younger women and poor men older women, which may have led to a high age at first reproduction among the wives of Poor men. Second, wealth may have affected the probability and timing of conception and successful pregnancy [30]. However, we found no difference in the length from marriage to the first pregnancy between the wealth classes, which suggests that energetic constraints in Poor mothers did not delay their first reproductive event. Third, Poor women might have delayed their reproduction through behavioural means in order to adjust their child number to match their unfavourable economic circumstances. Our data support this latter conclusion that sociological rather than physiological reasons were behind the later age at first reproduction in the Poor women. In summary, poverty may have forced the Poor women to start their reproduction at an older age, despite the high fitness pay offs of earlier age at first reproduction.

Rich mothers exhibited the highest fecundity and their offspring had the best survival. In many traditional societies unaffected by demographic transition, such as in the Gabbra pastoralists of Kenya, fecundity increases with wealth [31]. Furthermore, life-history analysis using optimality modelling predicts that in such conditions, poor quality individuals should not reproduce at a maximal rate in order to maintain enough resources like livestock to marry off their children [32]. Lower fecundity might thus result from selection on reproductive restraint and greater investment in the rearing of offspring already born (i.e., trading offspring quality not quantity), rather than from poor body condition *per se*. In the Rich wealth class, some noble women (although very few in our study population) might have also adopted the custom of hiring wet nurses to breast-feed their babies [33], resulting in shorter inter-birth intervals and higher fertility [34]. However, because offspring survival and fecundity were negatively associated in the Rich, wet-nursing unlikely explains these findings.

In contrast to the age at first reproduction, age at last reproduction was under stronger selection in wealthier mothers than in the Poor mothers. That is, the Rich and the Middle-class women gained more fitness benefits through continuing reproduction until older ages than the women in the Poor wealth class. The Poor mothers also delivered their last offspring earlier than the wealthier mothers. Hence, the wealthier mothers had longer reproductive lifespan to bear offspring than the poor mothers. Younger age at reproductive cessation in the Poor women might have however been adaptive, if reproduction in advanced age was especially risky for their health and survival, or if the Poor women achieved their desired family size sooner than other women. Alternatively, earlier age at last reproduction may have resulted from the scarcity of resources among the Poor women, restricting their reproduction into older ages. This conclusion is supported by our data, since the Poor women were not able to reproduce early even though it would have been particularly advantageous for their fitness. On the other hand, the highest correlation found between the age at first and last reproduction was in the Poor mothers, which may also indicate a more pronounced trade-off between early and late reproduction among these women. Finally, younger age at last reproduction may simply reflect the reduced survival of the poorest mothers later in life.

Longevity and particularly long post-menopausal lifespan have been found to increase the fitness of pre-industrial mothers [4], [7], but their relative importance on female fitness remains unknown. According to our path models, in all wealth classes female lifespan was under the lowest selection compared to the other maternal key life-history traits studied. We must note, however, that here the strength of natural selection on female lifespan was estimated using women who had survived to reproduce at least once. As we ignored early mortality, our selection estimates must thus underestimate the importance of female survival on her Darwinian fitness. After all, women have to first survive to maturity to reproduce. Among the Poor mothers, the importance of long lifespan on fitness, via later age at last reproduction, was most likely due to their higher mortality rates during reproductive ages. By contrast, among the Poor wealth class only, maternal longevity was not under direct selection through its effects via offspring survival.

Life-history theory predicts that if variation in resource availability is predictable, or depends on age, plasticity of reproductive allocation should be selected for [1]. If the population is subdivided by life-long access to resources, selection may lead to divergent evolution on life-history traits, as each wealth class has its own optimal life-history trait combination. Differences among the wealth classes in resource availability would then determine how substantial the differences in selection on life-history traits are, and gene flow between the wealth classes and heritability of life-history traits whether class-specific evolution of life-history traits is possible. In this study, we found evidence for differing selection pressures on female life-history traits between wealth classes. Furthermore, in a previous study investigating another population of historical Finns [24], we demonstrated significant additive genetic variation for many maternal key life-history traits, including fecundity, lifetime reproductive success, age at last reproduction and longevity, whereas age at first reproduction was mainly affected by family effects. This indicates that genetic variation allowing response to selection for these traits most likely existed. However, one probably needs to estimate heritabilities separately for each wealth class, since also the amount of additive genetic variance can differ between the environments [reviewed in 35]. A recent study on Soay sheep suggests that in poor environments, selection, for example, on survival may be stronger, but the amount of heritable genetic variation respectively smaller [35]. In other words, while animals may show high heritable variation in good environments, selection may be relaxed in these conditions [36]. This may explain why phenotypic trait means may not well correspond to (directional) selection acting on them. Finally, in our study populations half of the women differed in their wealth class compared to their mothers' wealth class. This indicates significant gene flow between the wealth classes at least through the female line that likely constrained diverging evolution between the wealth classes in our study populations.

In conclusion, this study provides the first estimates of opportunity for natural selection and the strength of directional selection on several female life-history traits according to the socio-economic status. In line with the predictions of the life-history theory, mothers of the poorest wealth class, who suffered from the highest age-specific mortality, had stronger selection on earlier age at first reproduction than on the age at last reproduction. Instead, selection for later age at last reproduction outweighed selection for earlier age at first reproduction in wealthier mothers. Our results also suggest that low resource availability among the Poor women constrained their ability to maximise fitness, for example, by starting reproduction earlier and increasing offspring survival. Compared to the poorer mothers, Rich women started their reproduction earliest, had the highest fecundity and reproductive success and, finally, the highest number of grandchildren born. Because the ample gene flow between the wealth-classes likely decreased the rate of divergent evolution between the wealth classes, it is also plausible that the phenotypic trait means between the wealth classes differed due to phenotypic plasticity. Nevertheless, our results demonstrate that resource availability was likely to affect the strength of natural selection on life-history traits and had an important role in shaping the life-history evolution of pre-industrial women.

## Materials and Methods

### Demographic Data

The influence of wealth class in modifying selection for female life-history traits was studied using demographic data collected from Finnish population registers of the pre-industrial era. The Lutheran Church has kept census, birth/baptism, marriage and death/burial registers of each parish in the country since the 17th century, covering the whole population of Finland from 1749 onwards. We used demographic data collected from five Finnish parishes (Hiittinen, Kustavi, Pulkkila, Rymättylä, and Ikaalinen) of the 18–19^th^ century [37]–[38]. We recorded complete life histories for mothers and for one generation of their all reproductive female offspring (*n*  =  704). During the study period these populations depended on farming and fishing for their livelihood [16], [27] and experienced high mortality and fertility due to the lack of modern medical care and contraceptive methods.

We classified individuals according to their socio-economic status. Because we had no direct knowledge of the actual wealth of the families, such as taxes paid or farm size, and since women at our study period rarely had an occupation of their own, we used a husband's occupation as a reference to wealth and social status of women. We divided women to three wealth classes; rich, middle-class, and poor. The Rich class included noblemen, priests and free farmers, the Middle-class included mainly tenant farmers and craftsmen, while the Poor included servants and dependent lodgers. This categorization was based on the historical studies of Finnish populations [37]–[38]. Inheritance of wealth class for females was moderately high: in these data, 54% of the Rich women's daughters had the same wealth class as their mothers. For the Middle-class and the Poor, the inheritance of wealth class was 62% and 39%, respectively.

We studied the following female life-history traits:

1. Age-specific probability of survival according to the wealth class of the parents
2. Probability of marriage by the wealth class of the parents for those women who survived to age of 20 years
3. Age at first reproduction (AFR), including illegitimate births
4. Time in months from marriage date to birth of the first child excluding women who had their first child before marriage
5. Fecundity (FEC), the number of children born to a woman during her lifespan
6. Offspring survival (%SURV), the proportion of children born that survived to age of 15 years
7. Age at last reproduction (ALR)
8. Lifetime reproductive success (LRS), the number of children who survived to age of 15
9. Longevity (LONG), age at death
10. Number of grandchildren born

Traits 3–9 included all women who had at least one child and for whom all studied life-history traits were known (*n* = 704).

### Statistical Analyses. *Survival*

We used Cox regression to examine how parental wealth class affected the age-specific survival probability of females [39]–[40]. Study parish and birth cohort were included into the model to take spatial and temporal variation in female survival rates into account. Assumption of proportional hazards was checked by including time-dependent covariates of explanatory variables into the initial model [40], but no evidence for time-dependence of these effects was found.

### Opportunity for selection and the strength of natural selection on female life-history traits

First, we estimated the overall constraint on total selection between the wealth classes by estimating the opportunity for selection (variance/mean^2^) on maternal fitness across wealth classes [41]. We also estimated the opportunity for selection on female fecundity and lifespan. The statistical significance of these estimates were tested by Levene's test.

Second, we studied the strength and direction of natural selection on female life history by estimating selection differential for traits using path analysis, performed on the variance-covariance matrix [26]. In the selection analyses, we used the residuals of traits obtained from generalized linear mixed models [42] in order to remove variation due to birth parish (correction of spatial variation), birth cohort of 20-year-intervals (correction of temporal variation), the effects of twin deliveries at any point of lifespan [20]–[21] and the effects of maternal family line (included as a random factor to correct for variation due to maternal effects) from fitness and all the life-history traits measured.

We started estimating the natural selection on female life history by constructing an *a priori* theoretical path model for the relationships between fitness and the female life-history traits measured (Fig. 2*a*). As a surrogate of fitness, we used the number of offspring raised to age 15 (LRS), which is shown to correspond well to the long-term individual genetic contribution to the future population gene pool [43]. In this model, ages at first and last reproduction were assigned to have a direct effect on fecundity, which, in turn, have a direct positive effect on fitness [4]–[5]. We also assumed a positive effect of longevity on fecundity, since the women's likelihood of having a large family size should increase with long lifespan. The proportion of offspring surviving to adulthood was assumed to have a direct positive effect on fitness and a negative effect on fecundity, since when a child dies as an infant, a mother should be more likely to become pregnant sooner [44]. We also assumed that age at first and last reproduction [5], age at first reproduction and longevity [45], and age at last reproduction and longevity [5] were correlated.

The estimated selection differential is the sum of direct and indirect selection on a trait relating to fitness [26]. Direct selection on a trait is estimated by its direct effect and effects through intermediate steps on fitness, whereas indirect selection on a focal trait is estimated by its effects via correlations with other traits related to fitness in the model [26]. It is not possible to test the statistical significance of a selection differential, if it involves intermediate steps to fitness or indirect selection [26]. Hence, statistical inference in path analysis is based on significant path coefficients and on the fit of the whole model to the data [26].

The fit of the path model was assessed by comparing the expected and observed covariance matrices by goodness-of-fit test, based on chi-square and comparative fit index (CFI) [46]–[47]. If CFI index exceeded 0.9, the fit of the model was regarded as acceptable [47]. Moreover, we used RMSEA-estimate (root-mean-square error of approximation), where an estimate <0.05 is considered to indicate a good fit and estimates >0.1 to be unacceptable [48]. The largest variance inflation factor was 1.9 and the smallest tolerance value was 0.5 for independent variables, indicating that multicollinearity was not a problem in path models [49].

We started the path analysis by estimating the *a priori* theoretical model (see Fig. 2*a*) for each wealth class separately and fitting this model for each wealth class separately. Selection on female life history was wealth class-specific, as indicated by the poor fit of *a priori* model in Rich (*χ2_5_* = 15.57, *P* = 0.008, RMSEA = 0.08) and Middle-class women (*χ2_5_* = 17.72, *P* = 0.0001, RMSEA = 0.1). Among Poor women only the fit of *a priori* model was acceptable (χ2_5_ = 2.28 *P* = 0.08, RMSEA =  0.00). This indicates fundamental differences among the wealth classes, and one should not continue by the assessment of parameter equalities to compare path coefficients between groups [50]. Instead, one should fit a different model for each group. We thus estimated a separate model for each wealth class, by sequentially excluding non-significant paths (*P*>0.05) from the models. Therefore, we estimated the strength of natural selection on female life-history traits separately for each wealth class, using final path models that remained after model reduction. As path coefficients, we present standardized selection gradients to make comparisons between wealth classes meaningful [51]. The final path models showed a reasonably good fit to the data among the Poor class only (Fig. 2), while in the Rich and the Middle-class, according to the chi-square test, the models did not fit the data well (Fig. 2*c*). As biologically meaningful modifications of these models did not improve the fit and because the RMSEA-value and CFI-index indicated an acceptable fit, we accepted these models as an appropriate description of natural selection in the Rich and Middle-class women.

The differences of path coefficients between wealth classes were examined by analysing interactions between wealth class and selected path coefficients using general linear mixed models (GLMMs) [52]. GLMMs controlled for study parish, birth cohort, and an effect of twin deliveries as fixed effects and maternal family line as a random effect. If significant interactions were found, we proceeded by conducting pairwise comparisons between wealth classes.

### Comparison of life-history traits between wealth classes

General and generalized linear mixed models [52] were used to assess whether parental wealth class affected a woman's probability of marriage, age at marriage, time it took from marriage to the first pregnancy, and whether family wealth class affected a woman's age at first and last reproduction, longevity, fecundity, proportion of offspring surviving to age of 15, lifetime reproductive success (LRS), and the number of grandchildren born. Maternal line was fitted as a random factor to account for the correlated measures of women who were sisters [42]. Logistic regression analyses investigating the effects of parental wealth class on marriage probability and the effects of marital wealth class on offspring survival were conducted by fitting the models using the generalized estimating equations (GEEs) with binomial errors and logit-link function and maternal family line as a random factor [52]–[53]. All the above analyses controlled for study parish and birth cohort. Least square means and their ±1 standard errors for these life-history traits are presented in Table 3. All analyses were performed with SAS analyses package (SAS version 9.1 Institute Inc.).
